# Utilizing a tablet-based artificial intelligence system to assess movement disorders in a prospective study

**DOI:** 10.1038/s41598-023-37388-3

**Published:** 2023-06-26

**Authors:** Maximilian Purk, Michael Fujarski, Marlon Becker, Tobias Warnecke, Julian Varghese

**Affiliations:** 1 Institute of Medical Informatics, University of Münster, Münster, Germany; 2 Department of Neurology and Neurorehabilitation, Klinikum Osnabrück–Academic Teaching Hospital of the University of Münster, Osnabrück, Germany

**Keywords:** Diagnostic markers, Movement disorders, Translational research

## Abstract

Spiral drawings on paper are used as routine measures in hospitals to assess Parkinson’s Disease motor deficiencies. In the age of emerging mobile health tools and Artificial Intelligence a comprehensive digital setup enables granular biomarker analyses and improved differential diagnoses in movement disorders. This study aims to evaluate on discriminatory features among Parkison’s Disease patients, healthy subjects and diverse movement disorders. Overall, 24 Parkinson’s Disease patients, 27 healthy controls and 26 patients with similar differential diagnoses were assessed with a novel tablet-based system. It utilizes an integrative assessment by combining a structured symptoms questionnaire—the Parkinson’s Disease Non-Motor Scale—and 2-handed spiral drawing captured on a tablet device. Three different classification tasks were evaluated: Parkinson’s Disease patients versus healthy control group (Task 1), all Movement disorders versus healthy control group (Task 2) and Parkinson’s Disease patients versus diverse other movement disorder patients (Task 3). To systematically study feature importances of digital biomarkers a Machine Learning classifier is cross-validated and interpreted with SHapley Additive exPlanations (SHAP) values. The number of non-motor symptoms differed significantly for Tasks 1 and 2 but not for Task 3. The proposed drawing features partially differed significantly for all three tasks. The diagnostic accuracy was on average 94.0% in Task 1, 89.4% in Task 2, and 72% in Task 3. While the accuracy in Task 3 only using the symptom questionnaire was close to the baseline, it greatly improved when including the tablet-based features from 60 to 72%. The accuracies for all three tasks were significantly improved by integrating the two modalities. These results show that tablet-based drawing features can not only be captured by consumer grade devices, but also capture specific features to Parkinson’s Disease that significantly improve the diagnostic accuracy compared to the symptom questionnaire. Therefore, the proposed system provides an objective type of disease characterization of movement disorders, which could be utilized for home-based assessments as well.

Clinicaltrials.gov Study-ID: NCT03638479.

## Introduction

Parkinson’s Disease is a widespread neurodegenerative disorder. The prevalence of PD in people over 60 years is about one percent^1^.

PD presents with complex, heterogenous symptoms. The primary characterization comes with the motor symptoms^2^, for instance a 4–6 Hz rest tremor, bradykinesia and muscular rigidity^3,4^. Because of variable manifestations, subtypes such as tremor dominant or hypokinetic types are described^5^. However, in early stages of Parkinson’s Disease, several non-motor symptoms exist such as loss of smell, constipation, depression and sleep disturbances^6^. Parkinson’s Disease with the above symptoms result in loss of quality of life and can reduce life expectancy^7^. Improving early diagnostics can improve quality of life through the timely introduction of therapy appropriate to the stage^8^. However, the heterogeneous manifestation implicates difficulty in diagnosis. The diagnosis of PD is a clinical diagnosis primarily based on the medical history and the clinical examination^9^. For staging and description, the clinic of the disease is often classified according to the Hoehn and Yahr scale, or the more comprehensive Unified Parkinson’s Disease Rating Scale (UPDRS)^10,11^.

The high prevalence combined with the poor rate of correctly diagnosed Parkinson’s Disease patients (PD) of 73.8% by general practitioners and 79.6% by movement disorder experts shows the importance of research in diagnosing movement disorders^12^. Furthermore, there is a need for objective and easy-to-use tools in fast track and telemedicine times as there is no reliable biochemical marker that is in daily use. Though, potential biomarkers are currently being researched at many different levels^13^.

In current research, digitalization is playing an increasingly important role. Some research groups are studying the voice of people with Parkinson’s as a subject of exploration^14,15^. In addition, there are experiments with wearables and analysis of the writing^16–18^.

The idea of analyzing tremors by a digitizing tablet was already published in 1990 by Elble et al.^19^, refined in several studies and used to classify between PD and healthy control or between PD and essential tremor^20–22^. Different types of tasks are analyzed such as writing letters or drawing a simple line^23,24^.

Memedi et al. built a machine learning classifier based on motor features that distinguishes between bradykinesia and dyskinesia^20^. The research group around Luciano et al. made another attempt with a spiral drawing examination to find an early biomarker in the diagnosis of PD. The reported sensitivity is 86% and the specificity 81%^25^.

Most previous studies found significant differences in the distributions of feature values between the different classes or promising results in machine learning. Nevertheless, there is no uniform device-based system recommended in diagnostics of Parkinson’s Disease^9^.

The research to date has tended to focus on the motor symptoms rather than the non-motor component^26^. Connecting both important feature types, motor symptoms (spiral drawing) and non-motor symptoms (questionnaire) in one integrative assessment provides promising potential for deeper disease characterization^27^.

The comprehensive approach of the Smart Device System with the inclusion of wearables and the studies of speech was extended to include spiral drawings^28^. The new data include the time, position and force values collected during the digitized spiral drawing for both arms and the Parkinson’s Disease Non-Motor Scale questionnaire (PD-NMS). As Chen et al. published, the best results in distinguishing different tremors are obtained if the participant follows a given spiral^22^. In order to include such an assessment in telemedicine, the examination procedure must be easy to implement^29^.

The first classification Task 1 aims to distinguish PD patients from a healthy control group having no known history of movement disorders (CG). The classification Task 2 aims to separate all movement disorders (MD) from the CG. Task 3 contains the most complex differentiation between PD and diverse movement disorders (DD) and is currently understudied^17^, as most previous work focuses on PD versus CG. Task 3 is of high clinical relevance, because the medical expert or the neurologist cannot assume whether the patient is either healthy or has PD. Therefore, disease features or classification models for potential diagnosis should be also evaluated against differential diagnoses to enable added medical value. In the era of digital transformation and mHealth, a tablet-based system enables simple integration highly of PD-relevant input, which are currently captured in hospital-based settings or assessment centers. The goal of the study is to capture spiral-drawing and questionnaire features and to evaluate the applied features in the context of these three tasks.

## Material and methods

### Study

**Figure 1:**
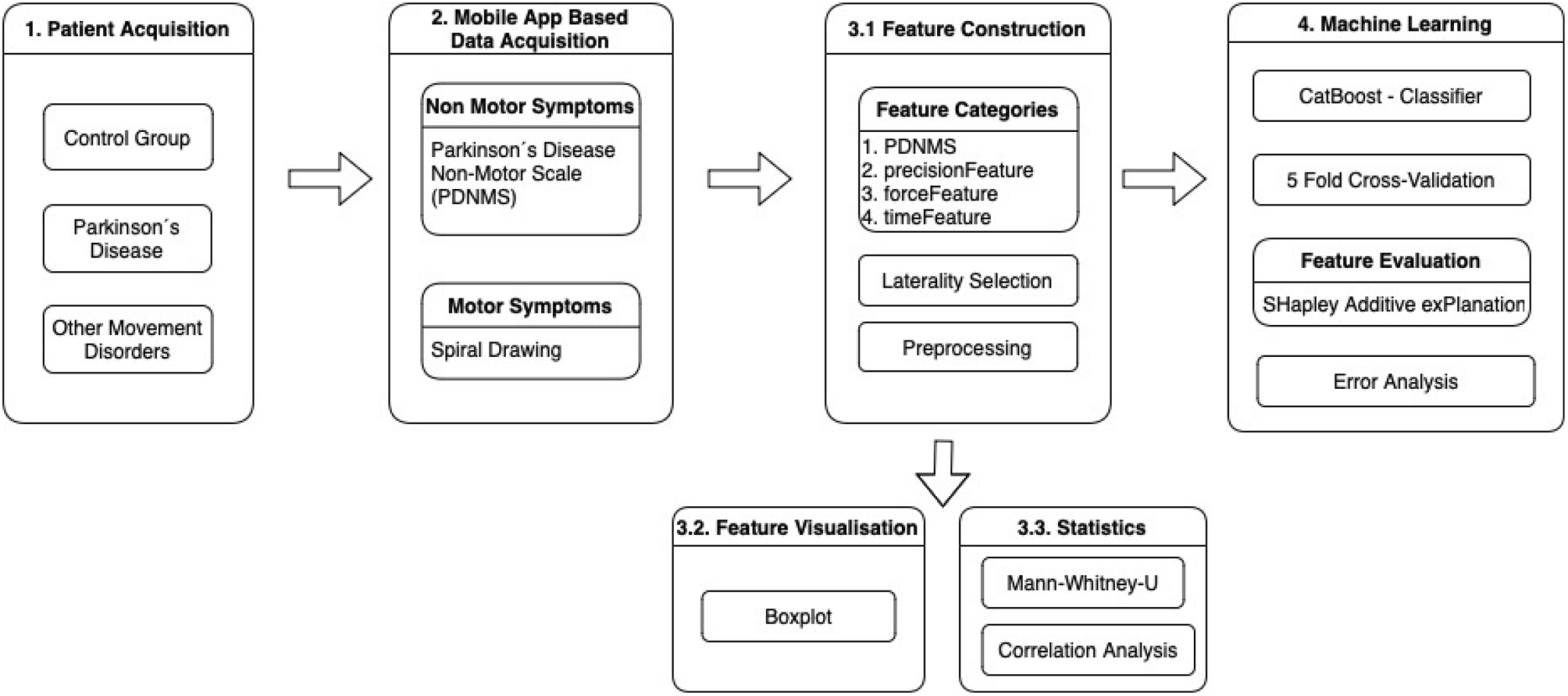
Study design in a flowchart.

The prospective study started in 2018 and was extended till the end of 2021. The methods were performed in accordance with relevant guidelines and regulations and approved by the ethical board of the University of Münster and the physician’s chamber of Westphalia–Lippe (Reference number: 2018.328.f-S). It was conducted at the outpatient clinic of movement disorders at the University Hospital Münster in Germany. The details of the study design and the protocol have been published previously^28^. Study registration ID on ClinicalTrials.gov: NCT03638479. All participants have provided written informed consent before participation. An overview of the study design can be found in Fig. 1. As a tertiary care center for movement disorders, there is broad access to patients affected by movement disorders at the outpatient clinic of the Department of Neurology at the University Hospital Münster. The DD class contains eight patients with essential tremor^30^, six patients with multiple sclerosis^31^, one tremor associated with lithium^32^, three patients with atypical parkinsonism^33^ (two multiple system atrophy and one progressive supranuclear paralyze), one tremor of unknown origin, one hand tremor associated with dystonia^34^ and two ataxia patients^35^. An overview of the demographic data can be found in Table 1. An assessment of the severity of PD can be made by indicating the distribution by the Hoehn and Yahr classification^10^.

**Table 1:** Demographic data.

	Control group (CG)	Parkinson’s disease (PD)	Diverse movement disorders (DD)
Number	27	24	26
Age: Median (IQR)	58.0 (22.0)	69 (12.0)	57.5 (17.25)
Gender: female (percentage)	10 (37.0%)	16 (66.7%)	13 (54.1%)
Hoehn and Yahr score^10^ Total count (percentage)	–	1: 3 (13%)	–
		2: 4 (17%)	
		2.5: 5 (21%)	
		3: 7 (29%)	
		4: 3 (13%)	
		5: 2 (8%)	

### Data acquisition

Details about the study design, procedures and preliminary results were published previously^28,36^. The examination was split into two parts. Information about the non-motor symptoms of the participants was captured by answering the Parkinson’s Disease Non-Motor Scale (PDNMS) as patient reported outcome. The 30 yes–no items included in the PDNMS check for typical non-motor symptoms of PD. Among them are details about their sleep, mood, sexual function and cognition^27^.

**Figure 2:**
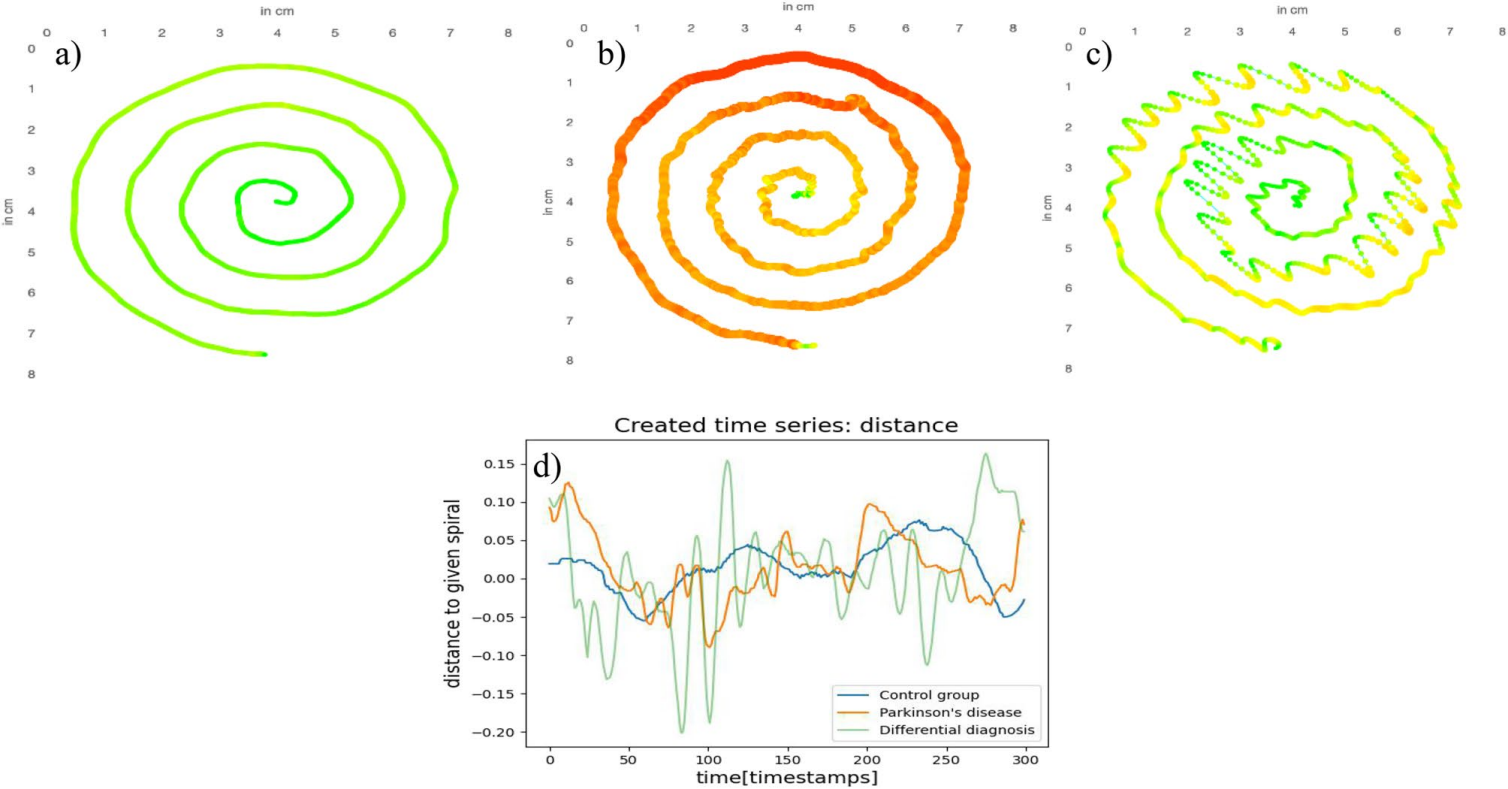
(**a**) Spiral drawing by CG study participant, (**b**) Spiral drawing by PD participant, (**c**) Spiral drawing by DD participant (Color: green: light force to red: strong force), (**d**) The first section of assessments from (**a**–**c**) visualizing the new time series distance showing the distance of the drawn spiral to the given spiral in cm. Negative values indicate a lower radius than the given spiral. The spirals of the PD and DD present with a higher variation.

The tablet-based assessment was designed with movement disorder specialists with more than a decade of experience in diagnosing and treating at the outpatient clinic for movement disorders. Participants were instructed to draw an Archimedean spiral twice with each hand. The spiral is to be drawn starting in the middle and then following the given lines with a stylus on a tablet. The maximal radius was 3.75 cm, and one spiral contains four loops (Fig. 2). The instructions to the test person did not involve further restrictions like time limits. This shall make potential homemade drawings comparable and decrease the dependency on the rater. As Kotsavasiloglou et al. propose, there is an advantage of basic assessments^23^. However, the loosely defined instructions introduce common errors that need to be addressed in later data processing. The recorded spirals were checked by the system whether they were drawn from the central point to the outside, otherwise the time series were reversed.

PD tremors are typically described as a unilateral tremor in the early stages^37^. By examination of only one hand, the one-sided tremor would not be captured for all cases. The two-handed approach observes the laterality of the disease. Executing the task for each side twice offers a more stable system against execution errors.

Data are acquired on an Apple iPad. The stylus used is an Apple Pencil which recorded the drawing with a sampling rate of 240 Hz. The raw data contain a multivariate time series containing a timestamp, x-coordinate, y-coordinate, and a force value for each data point.

### Features

Apart from the PDNM questionnaire, 13 variables representing motor symptoms were identified by reviewing comparable studies after searching for spiral drawing and PD assessment on PubMed and Google Scholar including grey literature from 2000 to 2022^22,24,38,39^. These features can be split into four categories. The first category contains the information of the PDNMS questionnaire and covers the non-motor symptoms^27^. The further three categories focus on the quantification of the motor symptoms by calculating a metric for precision, force, or time related. The features that address the motor symptoms will be numbered consecutively from F1 to F13. A list of all features can be seen in the supplements along with their category (S1).

The two corresponding feature values per arm were combined by the mean of the values. Since lifting the pen during the assessment is a common error, some outliers in the raw data needed to be filtered. In cases of time-independent features like the mean distance to the spiral, the top five percentage with the highest values were removed as outliers. For the time-dependent features the first and last 10 percent of the datapoints were clipped off due to high variance on dropping and lifting the pen.

The further analysis and feature extraction were performed on the participant’s arm with a more prominent tremor. In preparation, the x- and y-positions of the stylus were converted to a time series describing the distance to the given spiral. The side with the more significant standard deviation in the distance to the perfect spiral was considered the stronger affected side and these values were utilized for further analysis (Fig. 2).

### Non-motor symptoms features

QYes is the count of positive answered questions in the PDNMS described above. Non-motor impairments, such as cardiovascular or memory, were quantified by the PDNMS. The score includes a total of nine dimensions, including gastrointestinal symptoms and a subjective assessment of fatigue. Thus, the possible range is between 0 (all questions answered with “no”) and 30 (all questions answered with "yes")^27^.

### Precision features

The second group contains the features that quantify the precision of the spirals. It uses a method for generating the data regarding the distance to the given spiral. For F1c DistanceFFT, a discrete fast Fourier transformation (FFT) was applied on the distance-time series with the Python 3.8 NumPy package (version 1.20.2)^40^. The new data were reduced to the frequency spectrum between 3 and 15 Hz and quantized into 20 bins. On these bins, the standard deviation was calculated to detect the presence of a dominant frequency. A dominant frequency implies an overall low variation across the bins with a single outlier, and therefore a low standard deviation. For further analyses, the absolute value of the difference between both sides is calculated as information on laterality.

Feature F2 MaxDistance calculates the maximal distance for each drawn spiral to the given spiral. Features F3 MeanDistance and F4 StdDevDistance calculate the mean and the standard deviation of the given distance series respectively.

Feature F5 ChangeOfRadiusDirection counts the shifts of the radius from increasing to decreasing and vice versa. A perfect spiral has a steadily increasing radius. Introducing irregularities into the spiral, such as by tremor, increases the number of shifts. Features F6 ChangesOfDirectionX and F7 ChangesOfDirectionY are like F5 but only consider the X or Y axis respectively.

### Force features

The third group contains the force-related features. The force value provided through the Apple Development Framework is in relation to a predefined value with 1 equal to an average user input^41^. Features *F8 MeanForce*, *F9 StDevForce* and *F10 MedianForce* correspond to the mean, standard deviation and median of the applied force respectively.

### Time-related features

In the context of the bradykinesia and the rigor as potential motor symptoms in PD, the temporal dynamics of the drawing have to be analyzed^9^. The time-related aspect is considered in the last feature group. Feature *F11 TimeOfDrawing* calculates the total drawing time.

The x-, y-coordinates and time stamps are transformed into a time series of velocities. The velocity at each time instance is calculated according to (1).*f*, velocity function; *x*, *x*-coordinate; *y*, *y*-coordinate; *t*, time value.

1 $${f}_{i}({x}_{i}{,y}_{i},{t}_{i}) = \frac{\sqrt{{({x}_{i}{-x}_{i-1})}^{2}+{({y}_{i}{-y}_{i-1})}^{2}}}{({t}_{i}-{t}_{i-1})}$$

Calculating the discrete differentiation of the position with respect to the time and applying the Euclidean norm to acquire a 1-dimensional scalar for the absolute velocity at time instance *t*_*i*_. *f*_0_ is assumed to be 0.

Features *F12 MeanVelocity* and *F13 StdDevVelocity* are calculated as the mean or standard deviation of the velocity time series respectively (2).

*N*, total number of data points; *σ*^2^, variance; *x*, *x*-coordinate; *y*, *y*-coordinate; *t*, time value; *μ*, mean-value of $f_{i} \left( {x_{i} ,y_{i} ,t_{i} } \right)$

2 $$\sigma^{2} \left( {x_{i} ,y_{i} ,t_{i} } \right) = \frac{{\mathop \sum \nolimits_{i}^{N} \left( {f_{i} \left( {x_{i} ,y_{i} ,t_{i} } \right) - \mu } \right)^{2} }}{{\text{N}}}$$

Calculation of the variance of the velocity time series. Feature F13 equals the square root of the variance.

To improve the visualization, the log value of the drawing task-related features is used for further investigations since erroneous extreme values have less influence on the axis scaling.

### Feature statistics and visualization

**Figure 3:**
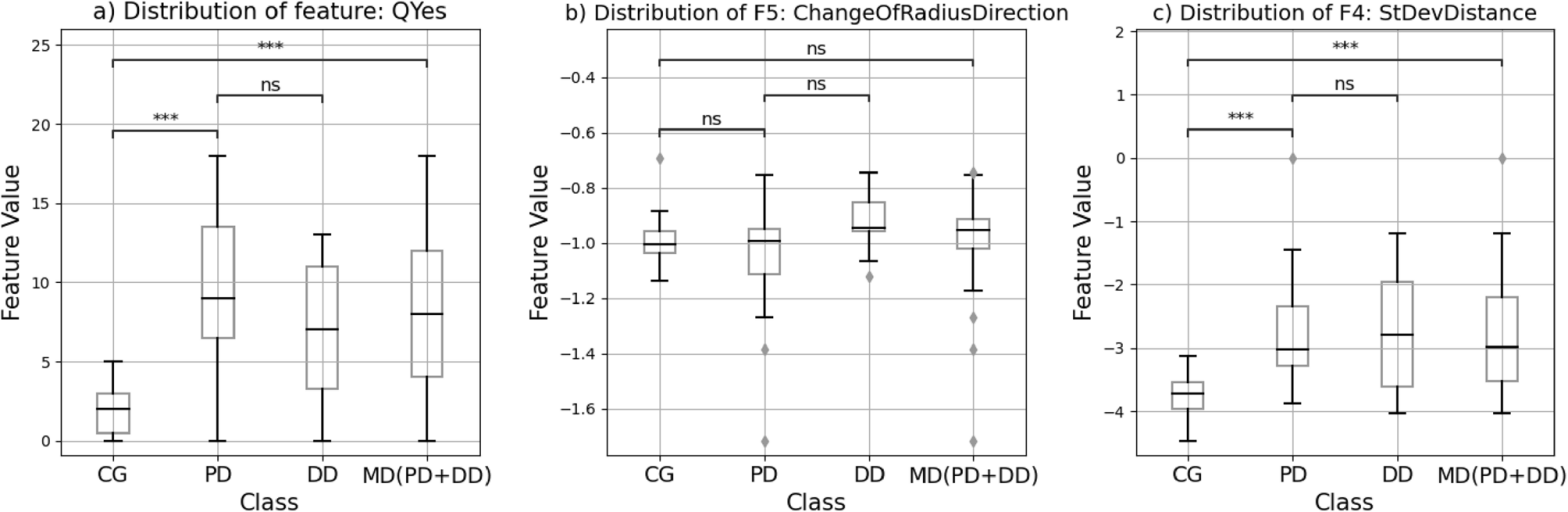
Boxplots for the three features. A Mann–Whitney-U test was conducted, *p*-value annotation legend after Bonferroni correction (*c* = 14): ns: *p* > 0.05/*c*; *: *p* ≤ 0.05/*c*; **: *p* ≤ 0.01/*c*; ***: *p* ≤ 0.001/*c*; ****: *p* ≤ 0.0001/*c*.

The feature statistics are split into two approaches. The Spearman correlation coefficient is used to determine correlations between the age, the feature values, and the tasks (Table 2) and the Mann–Whitney-U test is used to determine significant differences in means among the groups of Task 1, 2, and 3. *P*-values below 0.0036 were considered significant after applying Bonferroni correction. The correlations are calculated in Python 3.9.9 with the pandas 1.2.5 package^42^ while the Mann–Whitney-U was calculated using the Python package SciPy (version 1.6.3). The three features with high correlation with a classification target were chosen for testing for statistically significant differences between the classes (Fig. 3). A list of all tests is available in Supplements S4. For the Machine Learning cross-validation all features were used for training.

**Table 2:** Spearman correlation coefficient of the feature values, the age, and the classification task if the correlation is significant (*: *p* ≤ 0.05/*c*, **: *p* ≤ 0.01/*c*, ***: *p* ≤ 0.001/*c*, after Bonferroni-Correction *c* = 14).

	Age	Task 1 PD versus CG	Task 2 MD versus CG	Task 3 PD versus DD
Age	–			0.53**
QYes		0.63***	0.55***	
F1c DistanceFFT		0.44*	0.5***	
F2 MaxDistance		0.7***	0.58***	
F3 MeanDistance		0.66***	0.55***	
F4 StDevDistance		0.7***	0.58***	
F5 ChangeOfRadiusDirection				− 0.41*
F6 ChangeOfDirectionX				
F7 ChangeOfDirectionY				
F8 MeanForce				
F9 StDevDistance		0.46**	0.33*	
F10 MedianForce				
F11 TimeOfDrawing				
F12 MeanVelocity				
F13 StDevForce			0.36*

To examine the influence of the age distribution we calculated the correlation between the age and both, the feature values and the target variables as shown in Table 2. The correlation coefficients are listed for combinations with significant outcomes.

### Machine learning and SHAP analysis

**Figure 4:**
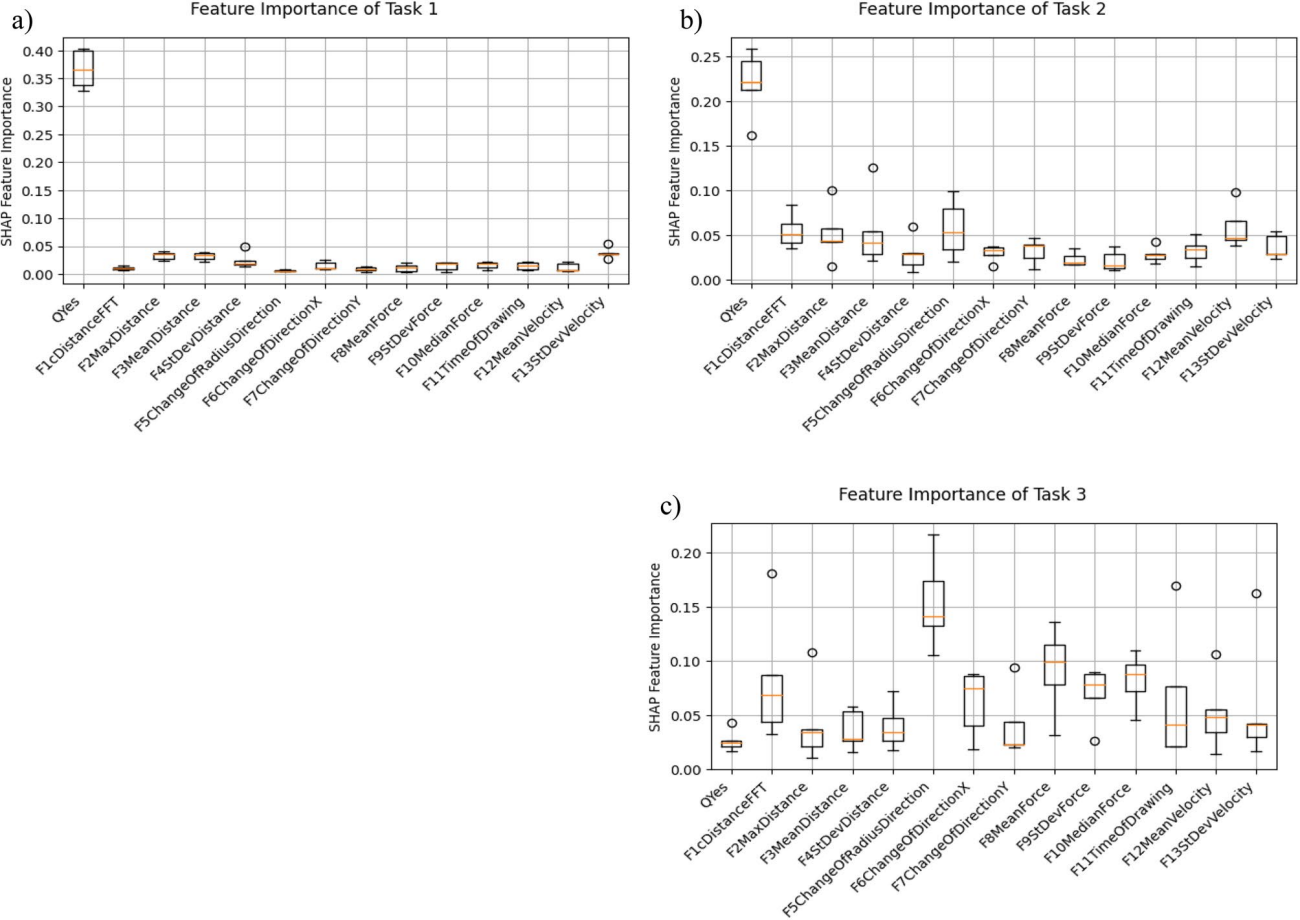
Results of the feature importance: Each boxplot represents the Mean SHAP value distribution of one feature over the fivefold cross-validation. (**a**) Task 1, (**b**) Task 2, (**c**) Task 3.

A CatBoost classifier is implemented and the accuracy, precision, recall and F1 for all three tasks are calculated to evaluate the potential of the classifier and features (Table 3). Furthermore, we included the accuracies for all three tasks for a majority class voter (dummy), a model only based on the PDNMS Score, and a model only based on tablet data to evaluate the benefits of integration. CatBoost was chosen because of the suitability with limited data and the high accuracy as an ensemble method^43^. The tablet features are normalized using a standard scaler and reduced in dimensionality with principal component analysis during the training phase. The default values are used as hyperparameters. To prevent overfitting, a stratified fivefold cross-validation was applied. For interpretability analyses, the Shapley Additive exPlanations (SHAP) values are used on the 5 resulting models (Fig. 4). The SHAP values describe the impact of the feature on the classifier outcome. Features with a high importance-variation show a high impact of the particular feature^44^. For further understanding, information can be found in the original paper by Lundberg^45^.

**Table 3:** Dummy: accuracy of simple majority class voting estimator.

	Task 1 PD versus CG	Task 2 MD versus CG	Task 3 PD versus DD
Dummy	0.53	0.65	0.52
QYes only	0.882	0.777	0.600
Tablet only	0.845	0.688	**0.700**
Integrated	**0.940**	**0.894**	**0.720**
Intgr. Precision	1.000	0.937	0.690
Intgr. Recall	0.880	0.900	0.740
Intgr. F1	0.928	0.916	0.706

QYes Only: Accuracy of estimator only using PDNMS Questionnaire. Tablet Only: Accuracy of estimator only using features acquired from proposed tablet-based assessment.

Significant values are in bold.

## Results

### Feature statistics and visualization

Table 2 shows the correlation between the target variable, the age, and the features. The tablet features partially have a strong correlation between the control group and PD as a distinct group (Task1) and the movement disorders combined (Task 2). The features correlate less to the differentiation of PD and DD (Task 3). The features *F1c DistanceFFT*, *F2 MaxDistance*, *F3 MeanDistance*, and *F4 StDevDistance* strongly correlate with Task 1 and 2 but are not significantly correlated with Task 3. Feature *F5 ChangeOfRadiusDirection* has a high anti-correlation with Task 3, low correlation with Task 2, and does not correlate with Task 1 at all.

There is no significant correlation between the features and the age distribution (Table 2). A full list of correlation coefficients regardless of the level of significance is available in Supplements S2. The highest correlation coefficient of the age is towards feature *F3 MeanDistance with a p-value of* 0.024. In addition, the age and Task 3 shows a positive correlation of 0.53.

The results of the statistical test are depicted above the boxes and indicate the level of significance. Similarly, to the correlation coefficients, the PDNMS score, and the distance-based tablet features significantly differ for Tasks 1 and 2, indicating potentially well-suited markers for detection of movement disorders. The Mann–Whitney U test did not show a significant difference in the distribution of any of the features between PD and DD.

### CatBoost score

The model was trained for three different feature sets for all three tasks as depicted in Table 3. The baseline dummy corresponds to a majority class voter. QYes Only and Tablet Only show the mean accuracy of the model trained on solely the PDNMS score and the tablet-based features respectively. Both subsets show an improvement in accuracy compared to the baseline. The PDNMS score performs better in Task 1 and 2 while the tablet features outperform the PDNMS score in Task 3. The integration of both data modalities greatly improves the performances for Task 1 and 2, while the PDNMS score only slightly improves the accuracy in Task 3.

### Feature importance

The high SHAP feature importance in Task 1 and 2 in Fig. 4 for the PDNMS questionnaire are consistent with the found correlations in Table 2. For Task 3 the questionnaire performs last, indicating only little impact on the differentiation of the considered movement disorders.

Higher absolute SHAP values suggest a higher influence on the model’s output. In Fig. 4a,b the *QYes* feature has the highest importance on the classifier. If more questions of the PDNMS were answered with "Yes" the more likely a disease becomes. For distinguishing MD and CG (Fig. 4), *F5 ChangeOfRadiusDirection* has the second highest impact followed by the mean and median force during the drawing (F8 and F10). In classification Task 3 PD versus MD (Fig. 4), the distribution for feature *F5 ChangeOfRadiusDirection* shows a high feature importance.

### Error analysis

To study the cause of misclassifications, we calculated the descriptive statistics for the erroneously classified subgroups for all Tasks in Table 4 for the tests sets of the cross validation. The PD patients that were not recognized in tasks 1 and 3 were on average older than 70 years. The false positives in Task 3 (DD identified as PD) were on average 52 years old and therefore younger than the false negatives (PD identified as DD).

**Table 4:** Error analysis descriptive statistics of false classifications.

	Task 1 PD versus CG		Task 2 MD versus CG			Task 3 PD versus DD	
	FP (0)	FN (3)	FP (3)	FN PD (0)	FN DD (5)	FP (8)	FN (6)
Age—Mean	–	72	56.3	–	48.6	52.1	70.7
Gender—Females	–	3/3	1/3	–	3/5	3/8	6/6
QYes—Mean	–	1.0	3.66	–	2.0	6.2	7.4
HaY—Mean	–	2.0	–	–	–	–	–

The age and gender distributions of the false positives and negatives in Task 2 were about the same. The PDNMS Score in Task 1 of the false positives was higher than the average CG with 3.6, while the false negatives had a significantly lower PDNMS Score with 1 on average. The same applies to the distribution in Task 2 with MD and CG. No PD patients were misclassified in Task 2.

## Discussion

Technology-based objective measure is emerging in the era of smart devices and this study shows the feasibility of objective drawings on a tablet and patient reported outcomes in distinguishing Parkinson’s Disease from healthy individuals and other movement disorders. While related work focuses on the former task of differentiating PD from healthy individuals^20,25^, our cohort includes a multitude of other movement disorders.

The Machine Learning classifier achieved an accuracy of 94.00% in Task 1 (PD vs. CG), 89.4% in Task 2 (MD vs. CG). Limiting the feature set to the tablet-based features results in an accuracy of 84.5% for Task 1 and indicates a slight improvement compared to similar work^20,25^. Task 3 (PD vs. DD) and performs with an accuracy of 72% which is comparable to the accuracy of non-expert physicians of 73.8% according to Rizzo et al.^12^. Cross-validation was employed as a rigorous method to assess the performance of the model on the available dataset and estimate its generalization performance. However, the performance of a model is influenced by the specific dataset used for training and testing, which could limit the generalizability of the findings. Therefore, it is necessary to conduct a future study with a larger dataset to enhance the transferability of the outcomes.

The novelty of the study is the combination and the integrated analyses of motor and non-motor symptoms assessments. The analysis of the motor symptoms based on drawing and the non-motor symptoms with the PDNMS resulted in noticeable information gain. The PDNMS shows high classification performance for Task 1 and 2, and a low performance close to the baseline in Task 3 (PD vs. DD). Therefore, the questionnaire is able to separate non-healthy participants from a healthy control group. This result is to be expected since the recruited MD patients include several neurodegenerative diseases that cause multiple non-motor symptoms. The tablet data performs slightly worse for Task 1, worse for Task 2, but considerably better for Task 3 compared to the PDNMS. The integration of the PDNMS and the tablet features improved the performance for all three Tasks. The features regarding global properties of the resulting spiral, like number of directional changes of the drawing, proved to be of no relevance for the classification tasks except for *F5 ChangeOfRadiusDirection* for Task 2 and 3. It describes the number of directional changes along the radial axis and indirectly measures the frequency of a possible tremor. The standard deviation of several properties has shown a significant impact on the classification of Task 1 and 2 and should be considered for further analyses of MD detection.

Including the tablet data significantly improves the performance for Task 3 compared to a simple questionnaire. Here, the proposed Feature *F5 ChangeOfRadiusDirection proved to be of* high feature importance. PD patients have significantly lower values, indicating that tremors for PD patients tend to have a lower frequency compared to patients of the other movement disorders group regardless of the amplitude.

However, the differentiation between PD and DD in Task 3 remains difficult. The versatility of symptoms of the recruited patients causes the PDNMS questionnaire to underperform^37^. Even the medication has a high impact on the symptoms^46^. The high variance in symptoms of the PD patients further makes it difficult to distinguish Parkinson’s Disease from other diseases^47^. The broad range in the Hoehn and Yahr Scale or the Unified Parkinson’s Disease Rating Scale demonstrates the high variability in manifestations^10,37^. The treatment with L-Dopa cannot prevent the switch between On- and Off States in later disease stages, which represents good motor function (On) and the immobility (Off)^9,48^. In the light of similar characteristics or pathogenesis of PD and diverse differential diagnoses as essential tremor, it remains challenging for the provided assessments to distinguish all these entities without further information, e.g., through clinical examination, imaging, or further biomarkers.

Due to the aging population, an increase in the prevalence of movement disorders is likely and the demand for objective diagnosis criteria based on cost-effective technology rises^49^. The results present the potential of the system’s method in health care, especially in settings with lack of movement disorder experts. There are chances of improving healthcare with telemedicine and maintain adequate health care in sparsely populated regions^50^. Moreover, the promising results of Task 2 to detect general movement disorders can provide the individual information about the need for an individual with motor abnormalities to visit the physician.

Next to the field of diagnosis, there is a broad area of usage of objective movement disorder assessments for monitoring the progress of symptoms. As mentioned in the introduction, there is a high disparity in the examination results between the physicians^12^. This tool makes individuals and their symptoms comparable, even over a long time and between different physicians.

In summary, there is potential in applying device-based tools that utilize sensors and patient reported outcomes for surveillance and diagnostics of movement disorders. Further research is necessary for finding disease-specific biomarkers to improve the classification of specific entities like Parkinson’s Disease from further differential diagnoses.

## Supplementary Information

Supplementary Information 1.

Supplementary Information 2.

Supplementary Information 3.

Supplementary Information 4.

## Data Availability

The data supporting our findings are openly available on our Git repository. https://imigitlab.uni-muenster.de/published/sds-tablet-based-ai.
