# Supplementary material for: Utilizing a tablet-based artificial intelligence system to assess movement disorders in a prospective study
Source: Sci Rep. 2023 Jun 26;13:10362. doi: 10.1038/s41598-023-37388-3 (PMC10293248; doi:10.1038/s41598-023-37388-3)
Supplement: Supplementary file 1 — Supplementary Information 1. [file 41598_2023_37388_MOESM1_ESM.pdf]

S1) A list of all used Features inluding their category. Tablet-based Features are split among precision-based, force-based, and time-based features.

Feature category	Feature ID	Feature Name	Preprocessing	Explanation
Questionnaire	Qyes	Qyes	None	*QYes* is the count of positive answered questions in the Parkinson Disease Non-Motor Scale questionnaire. Thus, the possible range is between 0 (all questions answered with "no") and 30 (all the answers are "yes").
Precision feature	F1c	DistanceFFT	Excision of first and last 10 percent	For F1 *DistanceFFT*, a discrete fast Fourier transformation (FFT) was applied on the distance-time series with the Python 3.8 NumPy package (version 1.20.2). The new timeseries was separated in the frequency spectrum between 3 and 15 Hz into 20 bins. On these bins, the standard deviation was calculated to see if there is a dominant frequency. In the case of a dominant frequency, a peak in the FFT data is observed corresponding to a low standard deviation. For further analysis, the absolute value of the difference between both sides is calculated to give information about monitored laterality.
	F2	MaxDistance	Excision of 5 percent most extreme values	Feature F2 *MaxDistance* calculates the maximal distance for each drawn spiral to the given spiral.
	F3	MeanDistance	Excision of 5 percent most extreme values	Feature F3 *MeanDistance* calculates the mean distance for each drawn spiral to the given spiral.
	F4	StDevDistance	None	Feature *F4 StDevDistance* calculates the standard deviation of the distance for each drawn spiral to the given spiral.
	F5	ChangeOfRadiusDirection	Excision of first and last 10 percent	Feature F5 *ChangeOfRadiusDirection* counts the times the radius goes from increasing to decreasing and the other way around.
	F6	ChangeOfDirectionX	Excision of first and last 10 percent	Feature F6 *ChangeOfDirectionX* counts the times the trend in the x-axis goes from increasing to decreasing and the other way around.
	F7	ChangeOfDirectionY	Excision of first and last 10 percent	Feature F7 *ChangeOfDirectionY* counts the times the trend in the y-axis goes from increasing to decreasing and the other way around.
Force feature	F8	MeanForce	Excision of 5 percent most extreme values	F8 *MeanForce* is the value the mean of the corresponding force timeseries.
	F9	StDevForce	None	F9 *StDevForce* is the value of the standard deviation of the corresponding force timeseries.
	F10	MedianForce	None	F10 MedianForce is the value of the median of the corresponding force timeseries.
Time feature	F11	TimeOfDrawing	None	F11 *TimeOfDrawing* discloses the total drawing time.
	F12	MeanVelocity	Excision of 5 percent most extreme values	F12 *MeanVelocity* gives information about the mean drawing velocity.
	F13	StDevVelocity	Excision of 5 percent most extreme values	F13 *StdDevVelocity* is the standard deviation of the drawing velocity time series.
