# Supplementary figures and images for: Utilizing a tablet-based artificial intelligence system to assess movement disorders in a prospective study
Source: Sci Rep. 2023 Jun 26;13:10362. doi: 10.1038/s41598-023-37388-3 (PMC10293248; doi:10.1038/s41598-023-37388-3)

## S2: Spearman correlations

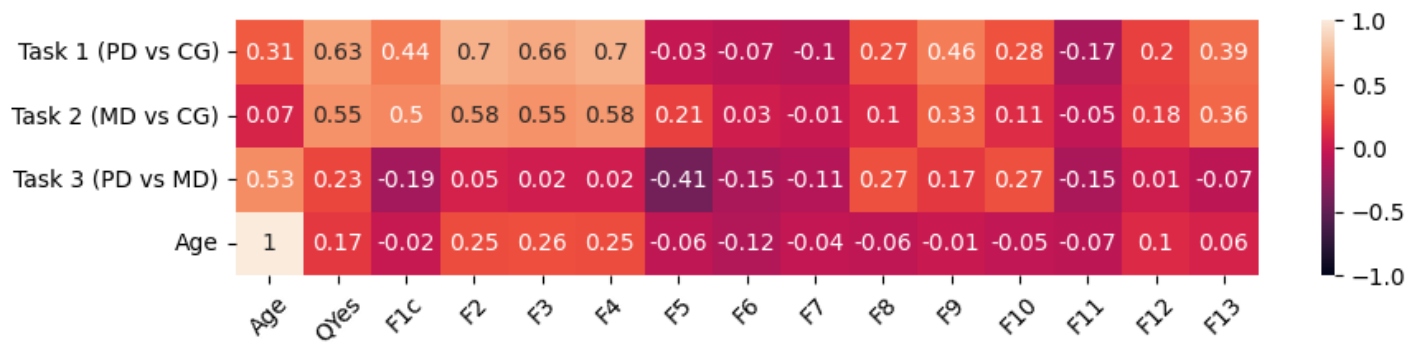

Supplement: Supplementary file 2 — Supplementary Information 2. [file 41598_2023_37388_MOESM2_ESM.pdf]
