# Supplementary material for: Utilizing a tablet-based artificial intelligence system to assess movement disorders in a prospective study
Source: Sci Rep. 2023 Jun 26;13:10362. doi: 10.1038/s41598-023-37388-3 (PMC10293248; doi:10.1038/s41598-023-37388-3)
Supplement: Supplementary file 3 — Supplementary Information 3. [file 41598_2023_37388_MOESM3_ESM.pdf]

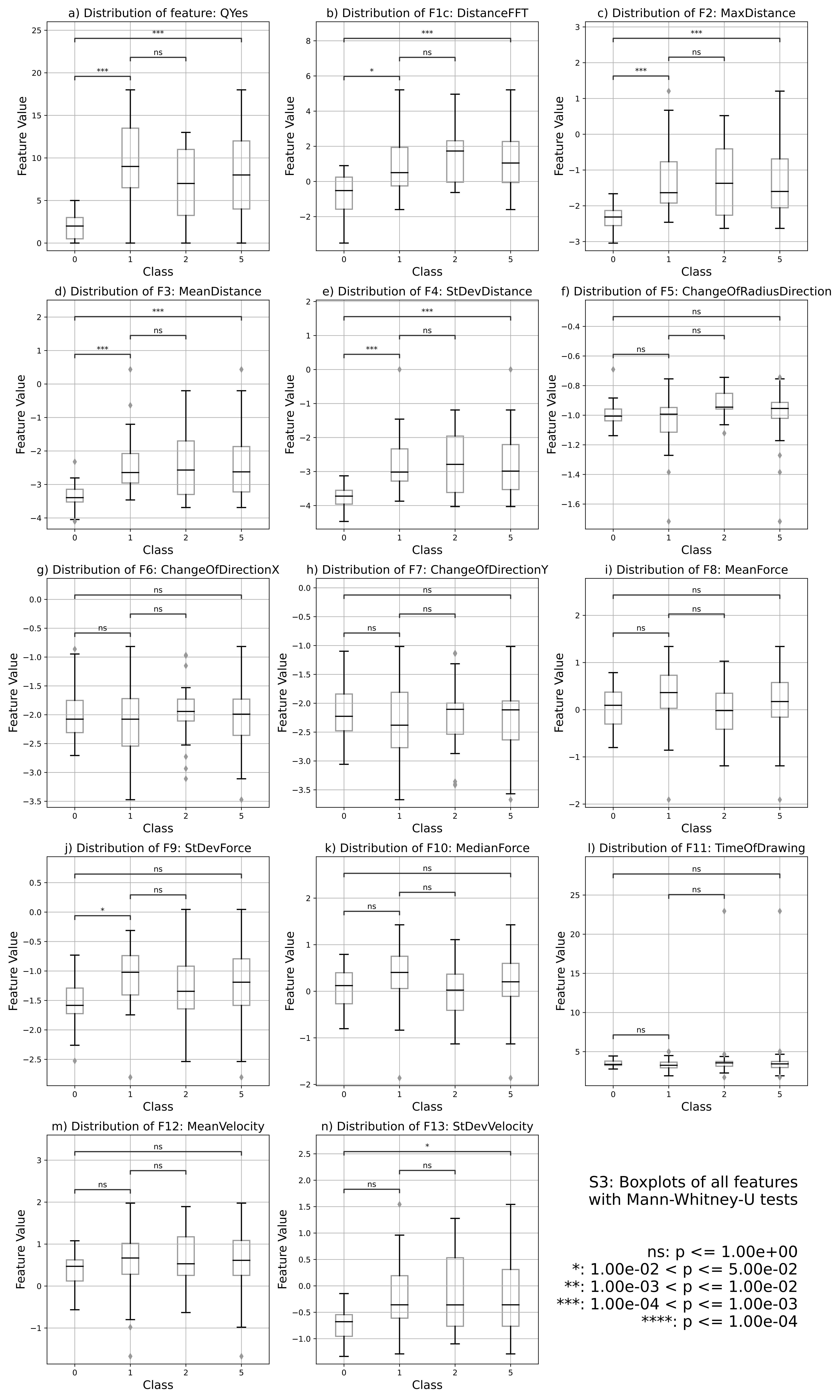
a) Distribution of feature: QYes
b) Distribution of F1c: DistanceFFT
c) Distribution of F2: MaxDistance
d) Distribution of F3: MeanDistance
e) Distribution of F4: StDevDistance
f) Distribution of F5: ChangeOfRadiusDirection
g) Distribution of F6: ChangeOfDirectionX
h) Distribution of F7: ChangeOfDirectionY
i) Distribution of F8: MeanForce
j) Distribution of F9: StDevForce
k) Distribution of F10: MedianForce
l) Distribution of F11: TimeOfDrawing
m) Distribution of F12: MeanVelocity
n) Distribution of F13: StDevVelocity
Feature Value
Class
S3: Boxplots of all features
with Mann-Whitney-U tests
ns: p <= 1.00e+00
*: 1.00e-02 < p <= 5.00e-02
**: 1.00e-03 < p <= 1.00e-02
***: 1.00e-04 < p <= 1.00e-03
****: p <= 1.00e-04
