# Supplementary material for: Utilizing a tablet-based artificial intelligence system to assess movement disorders in a prospective study
Source: Sci Rep. 2023 Jun 26;13:10362. doi: 10.1038/s41598-023-37388-3 (PMC10293248; doi:10.1038/s41598-023-37388-3)
Supplement: Supplementary file 4 — Supplementary Information 4. [file 41598_2023_37388_MOESM4_ESM.pdf]

S4 Results of a Mann-Whitney U test for all features for all three tasks

	Task 1 PD vs. CG	Task 2 MD vs. CG	Task 3 PD vs. DD
QYes	U = 88.5, p = < 0.001	U = 224.5, p = < 0.001	U = 230.5, p = 0.113
F1c: DistanceFFT	U = 158.0, p = 0.002	U = 263.0, p = < 0.001	U = 379.0, p = 0.193
F2: MaxDistance	U = 63.0, p = < 0.001	U = 202.0, p = < 0.001	U = 293.0, p = 0.712
F3: MeanDistance	U = 75.0, p = < 0.001	U = 225.0, p = < 0.001	U = 304.0, p = 0.877
F4: StDevDistance	U = 61.0, p = < 0.001	U = 203.0, p = < 0.001	U = 304.0, p = 0.877
F5: ChangeOfRadiusDirection	U = 335.0, p = 0.836	U = 506.0, p = 0.071	U = 458.0, p = 0.005
F6: ChangeOfDirectionX	U = 350.0, p = 0.624	U = 652.0, p = 0.806	U = 366.0, p = 0.294
F7: ChangeOfDirectionY	U = 362.0, p = 0.473	U = 682.0, p = 0.940	U = 350.0, p = 0.461
F8: MeanForce	U = 224.0, p = 0.059	U = 594.0, p = 0.387	U = 216.0, p = 0.062
F9: StDevForce	U = 151.0, p = 0.001	U = 404.0, p = 0.004	U = 250.0, p = 0.229
F10: MedianForce	U = 218.0, p = 0.045	U = 587.0, p = 0.348	U = 215.0, p = 0.060
F11: TimeOfDrawing	U = 387.0, p = 0.234	U = 718.0, p = 0.646	U = 366.0, p = 0.294
F12: MeanVelocity	U = 249.0, p = 0.157	U = 528.0, p = 0.117	U = 309.0, p = 0.954
F13: StDevVelocity	U = 178.0, p = 0.006	U = 381.0, p = 0.002	U = 338.0, p = 0.614
